# Metabolite-Driven Modulation of Biofilm Formation in *Shewanella*: Insights from *Shewanella* sp. Pdp11 Extracellular Products

**DOI:** 10.1007/s00248-025-02552-x

**Published:** 2025-05-27

**Authors:** Olivia Pérez-Gómez, Marta Domínguez-Maqueda, Jorge García-Márquez, Miguel Ángel Moriñigo, Silvana T. Tapia-Paniagua

**Affiliations:** Departamento de Microbiología, Facultad de Ciencias, Instituto Andaluz de Biotecnología y Desarrollo Azul (IBYDA), Universidad de Málaga, Ceimar-Universidad de Málaga, Málaga, Spain

**Keywords:** *Shewanella* sp. Pdp11, Extracellular products (ECPs), Biofilm inhibition, Metabolomic analysis, Glycogen

## Abstract

Biofilm formation is a survival strategy for bacteria, contributing to their persistence in natural and industrial environments. In this study, we investigated the ability of extracellular products (ECPs) produced by the probiotic strain *Shewanella* sp*.* Pdp11 under different culture conditions to inhibit biofilm formation in pathogenic and environmental *Shewanella* strains. ECPs from specific culture conditions altered biofilm formation in several *Shewanella* strains, with *Shewanella hafniensis* P14 displaying the highest sensitivity. Metabolomic analysis of the ECPs identified glycogen as a key metabolite associated with biofilm inhibition. Further genomic analysis of *S*. *hafniensis* P14 revealed an interruption in its glycogen synthesis pathway, suggesting a dependency on external glycogen-related metabolites for biofilm development. These findings demonstrate that *Shewanella* sp. Pdp11 ECPs can modify biofilm formation across multiple *Shewanella* strains, particularly in *S*. *hafniensis* P14 through glycogen-associated mechanisms.

**Supplementary Information:**

The online version contains supplementary material available at 10.1007/s00248-025-02552-x.

## Introduction

Biofilm formation is a widely recognized strategy employed by bacteria to enhance their survival in diverse and often hostile environments [1]. These structured microbial communities are embedded in a self-produced extracellular polymeric substance matrix, enabling adherence to both biotic and abiotic surfaces [2]. Biofilms provide bacteria with significant ecological advantages, such as increased resistance to desiccation, antibiotics and other environmental stressors, which pose considerable challenges in clinical, industrial and aquaculture systems [3]. In aquaculture, biofilms are of particular concern as they can act as reservoirs for opportunistic pathogens, leading to disease outbreaks, decreased water quality, and biofouling, ultimately compromising production efficiency [4].

The genus *Shewanella* represents a group of Gram-negative bacteria widely distributed in marine and freshwater ecosystems [5]. Members of this genus exhibit remarkable metabolic versatility, enabling their adaptation to fluctuating environmental conditions [6]. While some *Shewanella* species are recognized for their roles in bioremediation and electron transfer processes [7], others are implicated as opportunistic pathogens in humans, fish, and other aquatic organisms [8, 9]. In aquaculture, pathogenic *Shewanella* strains, such as *S*. *algae* and *S*. *putrefaciens*, are associated with infections and spoilage [10, 11], highlighting the need for strategies to control their persistence and biofilm formation.

Conversely, certain strains of *Shewanella*, such as *Shewanella* sp. Pdp11 (Pdp11), has demonstrated probiotic potential [12, 13], and has been shown to improve fish health by enhancing immunity and nutrient absorption when administered as a dietary supplement [14–17]. This strain produces extracellular products (ECPs) with antimicrobial, immunostimulatory and antiviral properties, making them promising candidates for use in aquaculture [18, 19]. The bioactive properties of its ECPs, which include enzymes, peptides and secondary metabolites, suggest their potential utility in modulating microbial communities and biofilm dynamics [20].

Despite the promising applications of Pdp11 ECPs, their precise mechanisms of action and the specific metabolites responsible for biofilm modulation remain poorly understood. Recent studies indicate that the composition of ECPs varies significantly depending on culture conditions, influencing their biological activity [21]. This variability underscores the need for a detailed investigation into the metabolomic profiles of ECPs and their functional roles in biofilm regulation. Understanding these mechanisms could provide novel insights into biofilm management strategies based on metabolite-mediated interventions.

The present study focuses on evaluating the effects of ECPs produced by Pdp11 under different culture conditions on the biofilm formation of several *Shewanella* strains, including pathogenic and environmental isolates. The rationale for selecting these strains lies in their ecological relevance and capacity to form robust biofilms. Pathogenic strains, such as *S*. *algae* and *S*. *putrefaciens*, pose threats to aquaculture due to their ability to colonize surfaces and resist standard treatments [8, 22]. Environmental strains, such as *S*. *hafniensis* and *S*. *baltica*, serve as models to explore the metabolic and genetic factors underpinning biofilm development in natural ecosystems [23, 24].

By integrating biofilm quantification assays, metabolomic profiling and genomic analyses, this work seeks to provide a comprehensive understanding of the interactions between *Shewanella* strains and the bioactive compounds present in Pdp11 ECPs. Evaluating how ECPs influence biofilm formation across different strains will not only elucidate the functional roles of specific metabolites but also highlight strain-specific responses driven by genetic and metabolic variability.

The findings of this study aim to expand our understanding of metabolite-driven mechanisms regulating biofilm formation. Such insights can pave the way for the development of sustainable, metabolite-based strategies to manage microbial communities in aquaculture and industrial applications.

## Methods

### Bacterial Strains and Culture Conditions

The strains used in this study were kindly provided by various research groups and are part of the collection available in our laboratory, which determined their inclusion in this work as representative models for evaluating biofilm formation in *Shewanella*. The probiotic Pdp11 (CECT 7627) was isolated in our research group from the healthy skin of gilthead sea bream (*Sparus aurata*) [13]. To analyze the effect of Pdp11 ECPs on biofilm formation, nine *Shewanella* strains were used in this study (Table 1). All the strains available in our laboratory were grown on tryptic soy agar plates (TSA) (Oxoid Ltd., Basingstoke, UK) supplemented with 1.5% NaCl (TSAs). The cultures were incubated at 23 °C for 24 h.

**Table 1:** *Shewanella* spp. strains used in this study

Characteristics	Specie	Strains	Origin isolation	Reference
**Fish probiotic**	*S*. *putrefaciens*	Pdp11	Gilthead sea bream skin	[25]
**Pathogenic**	*S*. *putrefaciens*	SH6	European eel ulcer	[8]
		SH16		
		SH9		
	*S*. *algae*	17960	Human skin ulcer	[22]
		18115948		
**Environment**	*S*. *hafniensis*	R1418	Flounder skin from the Baltic Sea	[23]
		P14	Cod skin from the Baltic Sea	

### Pdp11 Extracellular Products Production and Minimum Inhibitory Concentration Assay

Initially, one to two colonies of Pdp11 were inoculated into 10 mL of tryptic soy broth (Oxoid Ltd., Basingstoke, UK) supplemented with 1.5% NaCl (TSBs) and incubated at 23 °C for 36 h to reach the stationary phase. The extracellular products (ECPs) were collected following the method described by Liu [26], with modifications introduced by Domínguez-Maqueda [20].

The solid culture media used for Pdp11 ECP production included TSAs, and other media that enabled the production of ECPs with DNAse activity [21]: (A) 160 g/L of commercial aquafeed with 1.5% agar (F medium); (B) a medium containing 50 g/L of the microalgae-cyanobacteria mix (including *Chlorella fusca*, *Tisochrysis galbana*, *Microchloropsis gaditana* and *Arthrospira platensis*, in a 1:1:1:1 ratio) and 1.5% agar; or (C) 75% commercial aquafeed with 25% replaced by a mixture of microalgae-cyanobacteria mix (FM medium) with 1.5% agar. The aquafeed was provided by LifeBioencapsulation S.L. [20], and the specific composition of each medium was previously described [21].

**Fig. 1:**
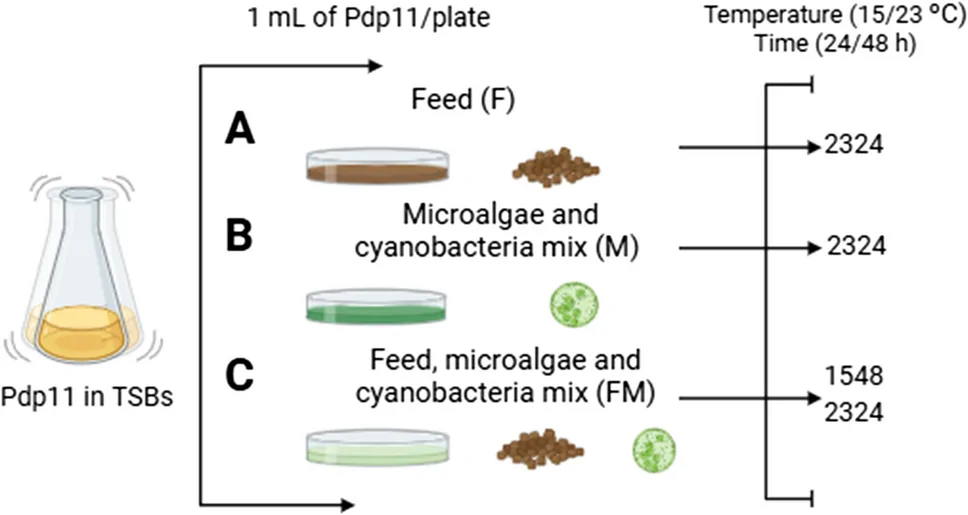
Schematic representation of the experimental setup for Pdp11 extracellular products (ECPs) production and DNAse activity previously described [21], which may degrade DNA, an essential component of the biofilm matrix. ECPs were extracted from F2324 (F plate at 23 °C for 24 h), M2324 (M plate at 23 °C for 24 h), FM1548 (FM plate at 15 °C for 48 h and FM2324 (FM plate at 23 °C for 24 h)

In all plates, 1 mL of Pdp11 culture or 1 mL of phosphate-buffered saline (PBS) for the internal control (IC) was spread onto the cellophane film. The IC served for testing small diffusible metabolites. All treatment and control plates were incubated under the same conditions: 23 °C for 24 h or 15 °C for 48 h. These media are summarized in Fig. 1.

After incubation, the ECPs were recovered by washing the plates with 5 mL of sterile PBS, followed by centrifugation (10,000 × g, 4 °C, 10 min). The supernatant was then filtered through a 0.22 µm, pore-size filter. The IC was obtained in the same way, but by collecting 5 mL of PBS from plates without bacteria.

ECP concentrations were equalized to 400 µg/µL based on total protein content, measured using the Qubit Protein Assay 2.0 (Thermo Fisher Scientific, USA). This normalization was applied to ensure consistent protein load across all ECP treatments. For all functional assays, including biofilm formation, ECP and IC concentrations were adjusted to levels below the determined minimum inhibitory concentration (MIC) values (see Supplementary Fig. S1), ensuring subinhibitory conditions.

The MIC of each ECPs produced by Pdp11 under different culture conditions was tested against nine previously described *Shewanella* strains. The assay was performed using 96-well microplates. Each well received a 1:1 dilution of Pdp11 ECPs (or culture medium as a negative control) mixed with double-concentrated TSBs. Twenty microliters of bacterial suspension adjusted an optical density (OD_595nm_ ~ 0.5) was added to each well. The microplates were incubated at 23 °C for 24 h. The MIC was identified as the lowest concentration of the ECPs that completely inhibited visible bacterial growth. ECPs concentration below the MIC was selected for further experiments, such as biofilm formation assays, to evaluate sub-inhibitory effects.

### Quantification of Biofilm Production Under Different ECPs from Pdp11 Cultured in Various Conditions Using Crystal Violet Staining

Then, biofilm formation assay was performed using the crystal violet (CV) staining method described by Vivas [27] with minor modifications [28]. Each strain was transferred to TSBs and adjusted to an optical density (OD_595nm_ ~ 0.5).

In the 96-well plate (#D51588, Sarstedt, Nümbrecht, Germany) biofilm assay, 90 µL of ECPs were added to each well along with 90 µL of 2 × TSBs, and 20 µL of bacterial suspension, reaching a final volume of 200 µL per well. This setup ensured a final ECP concentration (µg/µL) consistent with the sub-MIC threshold used across experiments.

The plates were incubated under static conditions at 23 °C for 24 h. After incubation, the medium was removed by inverting the plates, and the wells were washed three times with PBS to eliminate non-adherent cells. Biofilm layers were fixed with 200 μL of 99% methanol per well for 20 min. Excess methanol was removed, and the plates were air-dried. Wells were stained for 200 μL of 0.1% (w/v) CV solution for 15 min. Excess stain was washed out three times with PBS, and the plates were air-dried.

The CV stain was solubilized with 200 μL of 33% acetic acid per well. The plates were shaken at 200 rpm for 3 min at room temperature to release the dye. One hundred fifty microliters from each well was transferred to a new 96-well microplate, and the absorbance was measured at OD_595nm_ using a plate reader (Multiskan FC, Thermo Fisher).

Growth performance was evaluated in parallel with biofilm formation by measuring the optical density at 595 nm (OD595) prior to CV staining. This measurement allowed us to detect potential impacts of ECPs on planktonic cell growth. These OD values were normalized by subtracting the absorbance of wells containing only culture medium, and helped confirm that the biofilm differences observed were not simply due to changes in overall growth.

The results were used to identify the strain that produced the highest amount of biofilm, which was then selected for subsequent experiments to visualize biofilm structure. Additionally, metabolites derived from the ECPs that caused the most significant changes in biofilm production will be analyzed.

### Microscopic Analysis of Biofilm Structure and Morphology Using Confocal and Scanning Electron Microscopy Techniques

Based on the results obtained in the CV assay, *S*. *hafniensis* P14 was selected for biofilm microscopy analysis due to its high biofilm production and homogeneous morphology. Additionally, the ECPs of Pdp11 isolated from FM2324 were chosen for their significant impact on *S*. *hafniensis* biofilm formation. The biofilm structure was visualized using an inverted confocal microscope (Nikon-Eclipse Ti) equipped with phase contrast and fluorescence modules. Biofilm matrix staining was performed following an adaptation of the method described by Tan [29]. The biofilms were formed in 96-well plates after incubation for 24 h at 23 ℃, as previously described. The liquid medium was removed by inverting the plate, followed by washing with PBS. The biofilm was stained with Rhodamine B isothiocyanate mixed isomers (Sigma Chemical) dissolved in methanol at a concentration of 5 mg/mL. Excess dye was removed by washing with PBS, and the samples were air-dried in the dark. The fluorescence microscope revealed the attachment of Rhodamine B isothiocyanate to the exopolysaccharide matrix in the bacterial cell wall, allowing visualization of the biofilm structure. For image analyses, measurements were taken from eight distinct fields per sample.

Changes in the biofilm morphology induced by ECPs of Pdp11 were further examined using scanning electron microscopy (SEM). The samples were prepared following the methods previously described [30].

Subsequently, 60 μL of *S*. *hafniensis* P14 suspension was pipetted into flat-bottom polystyrene 24-well plates along with 270 μL of ECPs and filled up to 600 μL with double-concentrated TSBs. The microplates were incubated under static conditions at 23 °C for 24 h. After incubation, the biofilm was fixed by removing the culture medium and adding 200 μL ice-cold 3% (v/v) glutaraldehyde in PBS for 30 min at 4 °C. Plates were then rinsed with ddH_2_O and dehydrated using a graded ethanol series (55%, 60%, 70%, 80%, 90%, 95% and 100%) for 30 min each at room temperature. Ethanol was evaporated, and the bottoms of each well were cut into individual pieces using a hot lancet. Samples were coated with 4 nm platinum in an ion sputter (LEICA EM ACE600) and inspected by scanning microscope (FESEM TESCAN CLARA) at 15 kV. The images were captured in ultra-high resolution (UH-resolution) with a 10 µM scale. For SEM image analyses, measurements were taken from eight distinct fields per sample.

### Image Processing and Analysis

Images captured by confocal microscopy and SEM were processed using ImageJ software (National Institutes of Health, Bethesda, MD, USA) as described by [31]. Then, images were divided into red, blue, and green channels, and the red channel was selected. Images were converted to 32-bite grayscale, and the Threshold tool was used to differentiate the biofilm-covered area (BCA) (%) from the background (non-cover area) in 200 µm^2^ (scale: 2.55 pixel/µm).

SEM images were analysed to determine the number of bacterial cells and their length within a 20 µm^2^ (scale: 29.1 pixels/µm).

### Untargeted Metabolomics by UPLC-QTOF

Previous microscopy analysis results suggested the relevance of Pdp11 ECPs in the biofilm structure. The metabolomic analysis was carried out with the aim of deep-in of ECPs composition and its implication in biofilm regulation. To identify those metabolites characteristic of Pdp11 produced in FM2324 and FM1548 conditions, and absent in both internal controls (IC).

Triplicates of each ECP sample (150 mg) were extracted with 1 mL of methanol, vortexed for 5 min and centrifuged for 10 min at 10,000 rpm at 4 °C. After centrifugation, the supernatant was collected, filtered by 0.22 µm diameter and transferred to a vial for analysis. At the beginning, the instrument was calibrated to assure mass accuracy during the MS analysis using a mixture of reference compounds (Tuning Mix). Then, 2 µL of the sample was injected into the liquid chromatograph. The non-targeted metabolomics was using ultra performance liquid chromatography coupled to quadrupole time-of-flight (UPLC-QTOF) equipment as previously described [32].

Data were acquired using the Mass Hunter Workstation software and processed by Mass Hunter Qualitative Analysis software (version B.08.00, Service Pack 1, Agilent Technologies). As there were untargeted analyses, generic settings were applied to obtain as many compounds as possible. Each sample was injected into the group, and the order of sample injection was randomized to avoid sample bias. Besides, MeOH injections were included in every three samples as a blank run to avoid carry-over effects.

### Metabolite Identification and Statistical Analysis

Each peak was analyzed to identify one or multiple possible structures and to annotate metabolites using online databases and libraries, including MassHunter Personal Compound Database and Library (PCDL) (version B.08.00, Service Pack 1, Agilent Technologies), METLIN/ChemSpider/KEGG/Lipid Maps, PubChem, FAHFA and MDB database. Each candidate structure was scored based on its correlation with the MS/MS spectrum. Metabolites tentatively identified in libraries (score above 95%) were validated using standards. Tentative metabolites were selected for further validation based on their scores and biological significance in the study context.

The m/z, retention time and abundance for each ECPs condition were exported into a matrix using Mzmine 3 software. Tentative metabolites were then compared to the Human Microbial Metabolome Database (MiMeDB) (https://mimedb.org/) and selected for further validation based on their scores and biological relevance. Additionally, metabolites were grouped by class or subclass using the Yeast Metabolome Database (YMDB) (https://www.ymdb.ca/), and classified based on freely accessible chemical information of NCBI PubChem (https://pubchem.ncbi.nlm.nih.gov/).

Metabolomics assays were performed with 3 replicates of Pdp11 ECPs and 3 replicates as internal controls. For the analysis, ECPs obtained under the FM2324 condition were selected based on their effects on the biofilm formation of all *Shewanella* strains tested.

Statistical analysis of the data was performed using *t* tests and Wilcoxon rank-sum tests with MetaboAnalyst 6.0 (https://www.metaboanalyst.ca/) at a *P* value threshold of 0.05. Log_2_ (fold change, FC) and -log_10_ (*P* value) values were calculated and visualized on a volcano plot after false discovery rate (FDR) correction.

### Genetic and Functional Analysis of Glycogen Biosynthesis in Shewanella Species

#### Genomic Identification of Glycogen Biosynthesis Genes

Following preliminary insights from the metabolomic analysis results, glycogen was identified as a key metabolite potentially involved in biofilm synthesis and structure. To investigate the genetic basis for glycogen biosynthesis, protein sequences of glycogen biosynthesis enzymes described in Glg operon, including *glgB*, *glgX*, *glgC*, *glgA*, *glgP* [33]. The inability of *glgB*, *glgC* and *glgA* knockout mutants to accumulate glycogen has been previously reported [34]. Accordingly, the reviewed glgC, glgB and glgA protein sequences identified in *Shewanella* were retrieved from the UniProt database (https://www.uniprot.org).

To identify the presence of genes responsible for glycogen biosynthesis across the selected *Shewanella* strains, complete genomes were analyzed. For Pdp11, the complete genome sequence was obtained from the NCBI database (Accession: PRJNA312231, Biosample: SAMN04495905). For *S*. *hafniensis* (P14, R1418), *S*. *algae* (17,960 and 18,115,948) and *S*. *putrefaciens* (SH6, SH9 and SH16), genome sequences were referenced based on previously published studies [22, 23, 35]. When complete genome sequences were unavailable, genome assemblies available in the NCBI database were used (*S. putrefaciens*—taxid: 24, *S*. *algae*—taxid: 38,313, and *S*. *hafniensis*—taxid:365,590).

The presence and conservation of genes encoding glycogen biosynthesis enzymes were analyzed using TBLASTN version 2.14.0 + (https://blast.ncbi.nlm.nih.gov/Blast.cgi) to align retrieved protein sequences with the selected genomes. Coverage and identity thresholds were set to ≥ 80% and ≥ 90%, respectively, to confirm gene presence.

#### Functional Analysis of Glycogen and Raffinose in Biofilm Formation

To assess the functional role of glycogen in biofilm formation, bacterial cultures of *Shewanella* strains were supplemented with exogenous glycogen, molecular biology grade (Thermo Scientific). Biofilm assays were conducted following previously established protocols, with the addition of glycogen to the TSB medium. These metabolites have been reported to influence biofilm formation at low concentrations: glycogen (0.05 to 2%) [36, 37] and raffinose (0.0005 to 1%) [38, 39]. In this study, we used 2% glycogen and 1% raffinose, representing the upper range of concentrations previously described.

Control samples were cultured in TSBs, while experimental samples were grown in TSBs supplemented with glycogen or raffinose. Biofilm formation was quantified using CV staining after 24 h of incubation at 23 °C (see CV assay section). The assay experiments were conducted in triplicate, with five wells per strain (*n* = 5) in each assay. The effects of glycogen supplementation on biofilm structure and quantity were analyzed and compared to control samples.

### Statistical Analysis

The Shapiro–Wilk test was used to assess the normality of the data. Statistical analyses of bacterial growth, biofilm fluctuation (via CV staining), biofilm-covered area (BCA), bacterial count, bacterial length and glycogen assays were performed using one-way ANOVA followed by Dunnett’s multiple comparisons test in GraphPad Prism 8.0.1.

## Results

### MIC and Effect of Pdp11 ECPs on Biofilm Formation

Bacterial growth and MIC were determined (Supplementary Table S1). For the biofilm assays, the concentrations of ECPs and internal controls (ICs) were adjusted to values below the MIC thresholds (as indicated by the blue arrow in Supplementary Fig. S1).

**Fig. 2:**
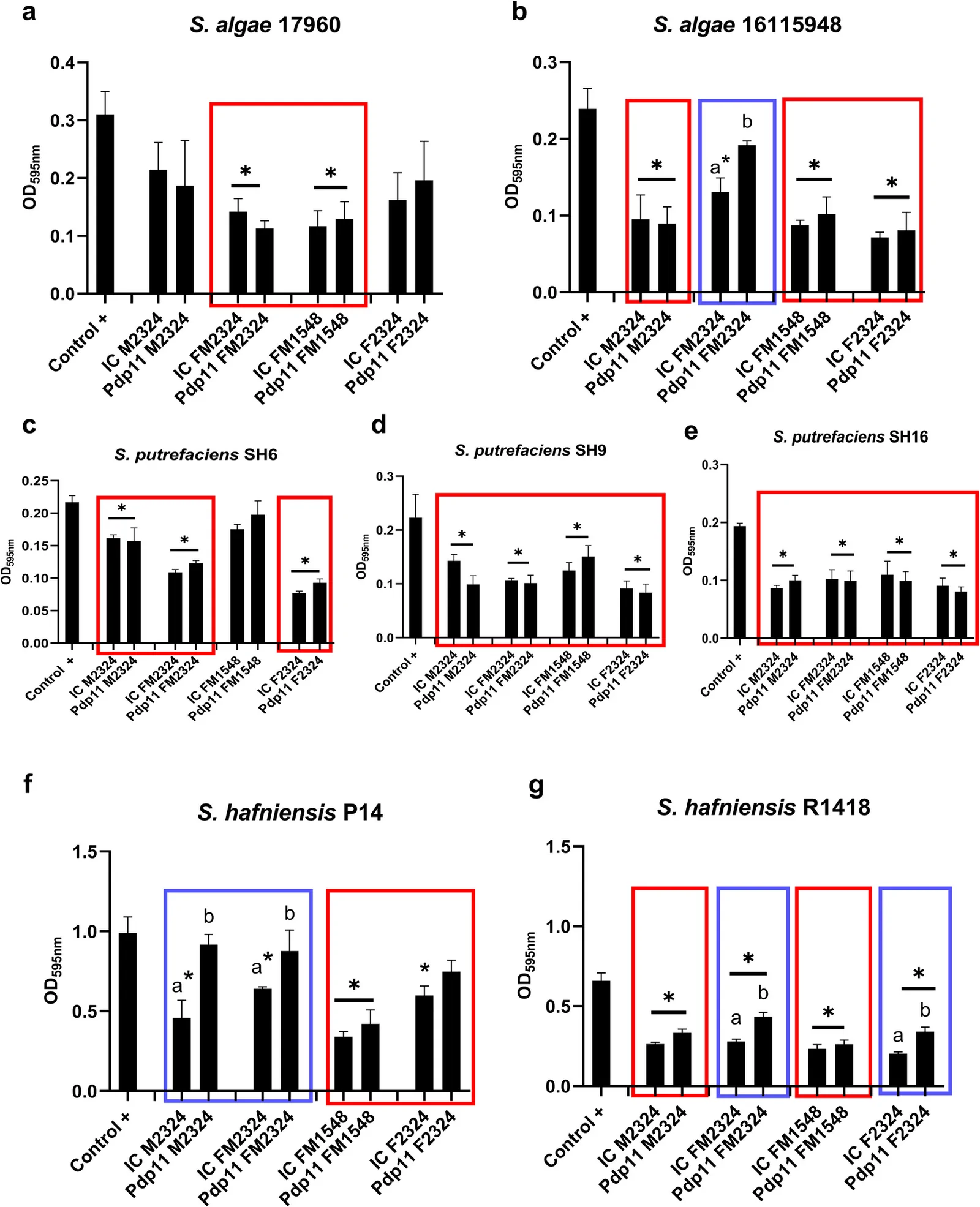
Biofilm production in *Shewanella* species. Amount of biofilm produced by *S*. *algae* (**a**, **b**), *S*. *putrefaciens* (**c**, **d**, **e**) and *S*. *hafniensis* (**f**, **g**) strains in response to Pdp11 ECPs and the internal control (IC) obtained from different culture media: aquafeed (F), microalgae-cyanobacteria mix (M), and aquafeed and microalgae-cyanobacteria mix (FM). Incubation conditions included variations in temperature (15–23 °C) and time (24–48 h), represented as F2324, FM2324, M2324 and FM1548. The control + represents the bacterial biofilm formation in standard TSBs medium at 23 °C for 24 h without ECPs or IC. Changes in biofilm formation are indicated with red (decrease) or blue (increase) boxes. Data are expressed as mean ± SD (*n* = 4). Squares with * indicate significant reductions in biofilm formation respect to Control + (*P* < 0.05). Different letters indicate significant differences between ECPs conditions and their respective IC (*P* < 0.05)

In general, *S*. *hafniensis* P14 and R1418 exhibited the highest levels of biofilm formation, with OD_595nm_ values of 0.8 and 0.6, respectively (Fig. 2(f–g)), compared to the other *Shewanella* species tested, which displayed OD_595nm_ values ranging between 0.2 and 0.3 (Fig. 2(a–e)), as determined by the CV technique. Pdp11 ECPs and the IC obtained from different media influenced the amount of biofilm produced by the tested *Shewanella* strains (Fig. 2(a–g)).

Notably, significant differences were observed between the IC and Pdp11 ECPs obtained from the different culture media. In general, both Pdp11 ECPs and IC significantly inhibited biofilm formation in *S*. *algae* strains 17,960 and 18,115,948, as well as *S*. *putrefaciens* strains SH6, SH9 and SH16 (Fig. 2(a–e)). However, ECPs obtained from the FM2324 condition promoted biofilm formation in *S*. *algae* strain 18,115,948 compared to the IC FM2324 (Fig. 2(b)).

Furthermore, ECPs from Pdp11 M2324 and FM2324 enhanced biofilm formation in *S*. *hafniensis* P14 compared to the IC (Fig. 2(f)). Similarly, ECPs from Pdp11 FM2324 and F2324 increased biofilm formation in *S*. *hafniensis* R1418 relative to the internal control (Fig. 2(g)).

### Effects of Pdp11 ECPs on Biofilm Structure

Based on the CV assay results, the Pdp11 FM2324 condition was identified as the one that affected all strains and was therefore selected for further biofilm structure analysis. Since *S*. *hafniensis* P14 exhibited the highest levels of biofilm formation, it was selected for microscopy analysis to evaluate the effects of ECPs on biofilm structure.

**Fig. 3:**
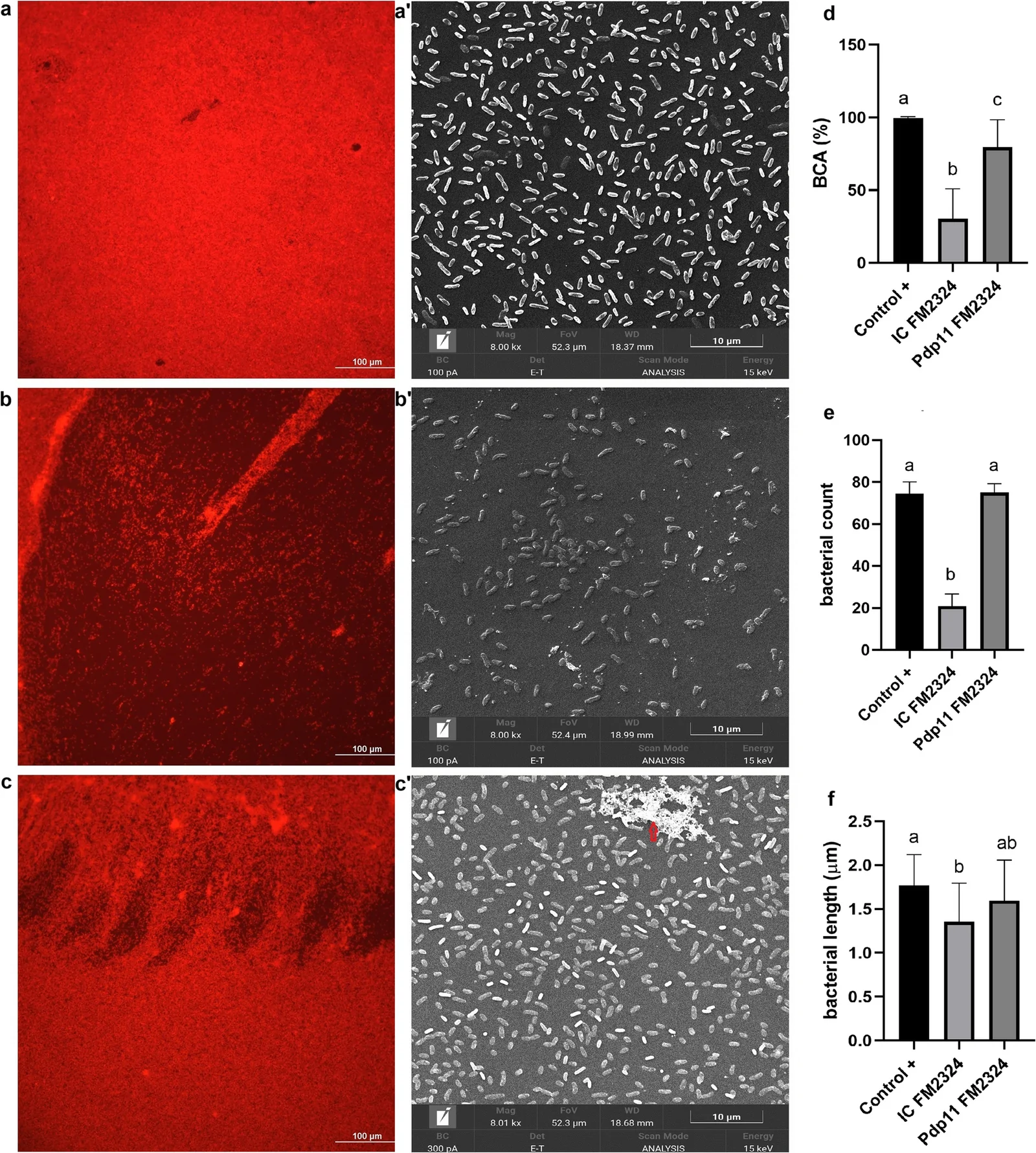
Biofilm structure of *S*. *hafniensis* P14 visualized by fluorescence microscopy (**a**, **b**, **c**) and SEM (**a′**, **b′**, **c′**) at 10 µm resolution. Biofilm morphology was analyzed under control conditions (**a**, **a′**), in contact with the IC from FM2324 (**b**, **b′**), and treated with Pdp11 ECPs from FM2324 (**c**, **c′**). Incubation was performed at 23 °C for 24 h. Biofilm-covered area (BCA), bacterial count and length (µm) were quantified (**d**, **e**, **f**). Different letters indicate significant differences (*P* < 0.05)

Under control conditions, the biofilm formed a homogeneous layer covering the bottom of the well (Fig. 3(a, a′)). Fluorescence imaging highlighted the biofilm layer (Supplementary Fig. S2), while SEM revealed rod-shaped bacteria (1–2 µm) embedded in an extracellular matrix (Fig. 3(a′)). Exposure to the IC from FM2324 media resulted in significant structural changes. Large gaps (> 400 µm) and areas of reduced bacterial density were observed (Fig. 3(b)), which correlated with a decrease in biofilm-covered area (BCA) (Fig. 3(d)). SEM analysis (Fig. 3(b′)) confirmed reductions in bacterial count (Fig. 3(e)) and length (Fig. 3(f)). Conversely, treatment with Pdp11 ECPs from FM2324 media restored biofilm integrity. These samples exhibited a more uniform biofilm matrix with smaller gaps and higher BCA compared to the IC (Fig. 3(c)). SEM imaging (Fig. 3(c′)) further supported these findings, showing bacteria embedded in a denser extracellular matrix (red arrows). Quantitative data revealed increases in bacterial count and length when exposed to Pdp11 ECPs.

### Metabolomic Profiling of Pdp11 ECPs and Their Impact on Biofilm Modulation

**Fig. 4:**
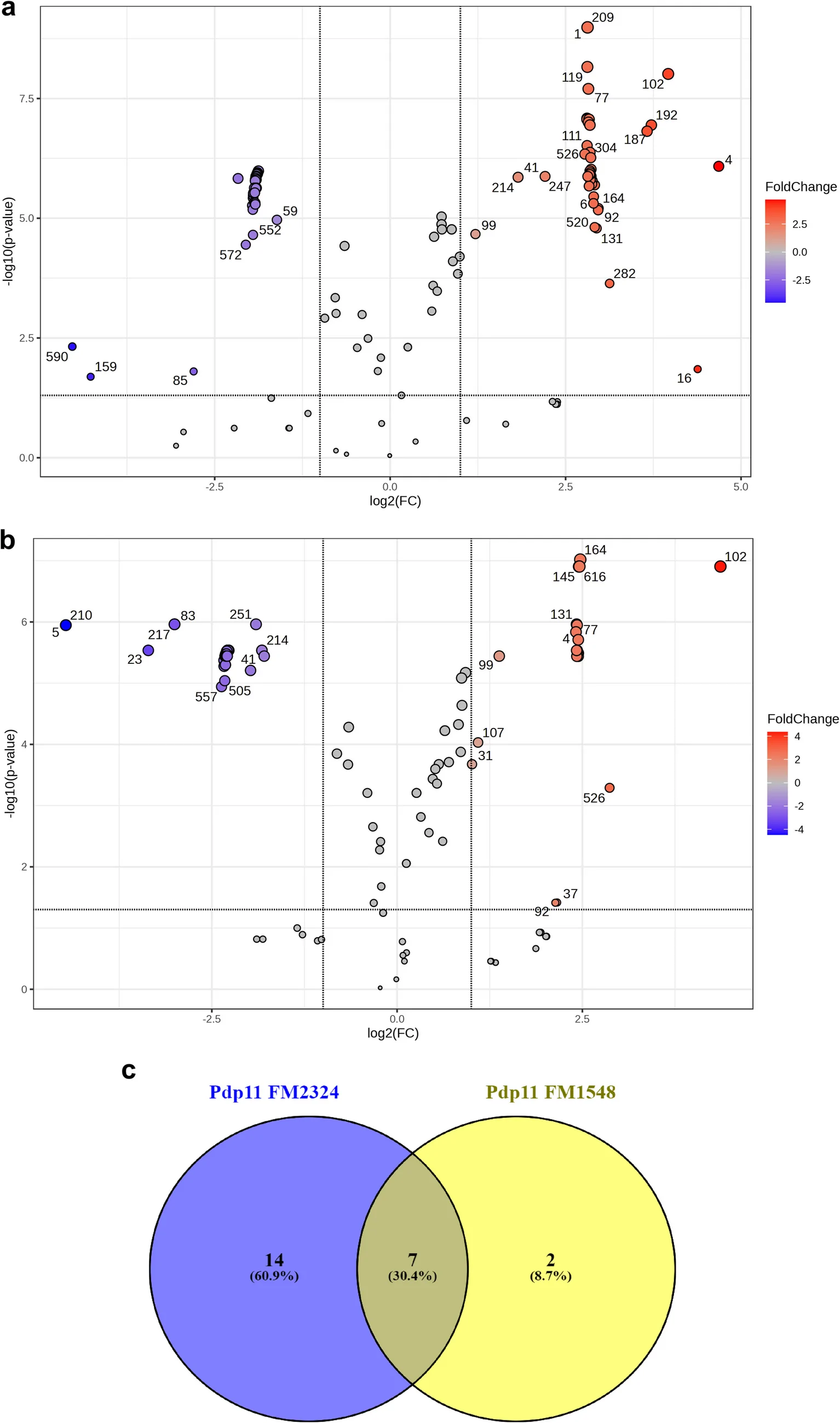
GC–MS plot of features obtained from Pdp11 ECPs. Volcano plots of (**a**) Pdp11 FM2324 and (**b**) Pdp11 FM1548. The x-axis shows log_2_/fold change, the y-axis shows − log_10_ (adjusted *P* values) by FDR correction. The dotted horizontal line denotes -log_10_ of our significance threshold (*P* ≤ 0.05), while the two dotted black vertical lines represent a -log_2_ of 0.5–2 for the fold change. Points represent non-significant metabolite (grey) changes, contrasting with the significance of up (red) and down (blue). The graphics reveal differences in a range of probiotic metabolites compared to their internal controls. (**c**) Venn diagram illustrates metabolites specific to Pdp11 isolated from FM2324 and FM1548 culture conditions, and absent at their internal controls

Metabolomic profiling of Pdp11 ECPs obtained under two culture conditions: FM2324 and FM1548 (which do not produce biofilm changes) revealed distinct metabolic signatures associated with biofilm modulation. Volcano plot analysis identified 148 metabolites as statistically significant (*P* < 0.05, |log2 FC|≥ 1), with 89 metabolites enriched in FM2324 (Fig. 4(a)) and 59 metabolites in FM1548 (Fig. 4(b)). Of these, 44 and 20 metabolites were upregulated in FM2324 and FM1548, respectively. Glycogen (ID 1/209) was particularly abundant in FM2324, correlating with increased biofilm formation in *S*. *hafniensis* P14 (see CV assay results). d-erythrose and taurine were also enriched under FM2324, while 6-acetyl-d-glucose and *N*-acetyl-d-glucosamine were predominant in FM1548.

A Venn diagram (Fig. 4(c)) represents the upregulated and bacterial-exclusive metabolites (absent at their internal control), which revealed that FM2324 and FM1548 shared 7 common metabolites (30.4%), while 14 metabolites (60.9%) were unique to FM2324, and 2 metabolites (8.7%) were exclusive to FM1548. Functional classification of these metabolites using YMDB and PubChem grouped them into key categories (Table 2), including glycogen in FM2324, amino acids, sulfur compounds, sugars, carbohydrates and nucleotides.

**Table 2:** Significative metabolites were identified at ECPs of Pdp11 FM2324, FM1548 or both culture conditions, which were absent at their culture media control (IC-FM2324 and IC-FM1548). The metabolites were classified by The Yeast Metabolome Database (YMDB)(*) or collection of freely accessible chemical information of NCBI (PubChem)(**)

YMDB^*^ or PubChem^**^ classification	Volcano ID	Annotation	Condition
*Amines***,** *Amino acids***,** peptides and analogues^*^, N-acyl-amino acid^**^	131	d−4′-Phosphopantothenate	FM2324 & FM1548
	148	*N*-Acetyl-l-Histidine	FM2324 & FM1548
	590	*N*-Acetyl-d-glucosamine	FM1548
	21 and 211	l-Methionine	FM2324
	282	l-Histidine	FM2324
	304	l-Lysine	FM2324
	33	l-Alanine	FM2324
	532	Phenylalanyl-Glycine	FM2324 & FM1548
	239	Methionyl-Glycine	FM2324
	509	DL-Histidinol	FM2324
	164	N2-Succinyl-l-arginine	FM2324 & FM1548
	180	l-a-glutamyl-l-Lysine	FM2324
Sulfur compounds^**^, Organosulfonic acids and derivatives^*^	97	3-Sulfinoalanine	FM2324
	108	Taurine	FM2324
*Sugar* **(***monosaccharides and oligosaccharide***)** polysaccharide^**^	289	d-Erythrose	FM2324
	17	Raffinose	FM2324
	616	6-Acetyl-d-glucose	FM1548
	1 and 209	Glycogen	FM2324
Purines and purine derivatives^*^	77	Guanine	FM2324 & FM1548
	34	Thioguanine	FM2324
Carbohydrates and carbohydrate conjugates^*^	26	5-Amino-6-(5′-phosphoribitylamino) uracil	FM2324 & FM1548
Carboxylic acid anion ^**^	119	2,5-Dioxopentanoate	FM2324 & FM1548
Glycerophosphoethanolamines^*^	107	Glycerylphosphorylethanolamine	FM2324

### Identification of glgC and glgB Genes in Shewanella Species

Although no glgA protein is listed as a reviewed entry for *Shewanella* in the UniProt database, we identified 12 sequences of glucose-1-phosphate adenylyltransferase (GlgC) and 10 sequences of glycogen branching enzyme (GlgB) classified as reviewed proteins within the genus (see Supplementary glgC and glgB FASTA files). Since the genomes of *S*. *putrefaciens* (SH6, SH9, and SH16), *S*. *algae* (16,115,948 and 17,960) and *S*. *hafniensis* (P14 and R1418) are not yet publicly available, a TBLASTN analysis was conducted using GlgC and GlgB protein sequences from *Shewanella* against all available genomes of *S*. *putrefaciens*, *S*. *algae* and *S*. *hafniensis* in the NCBI database. This approach enabled us to determine the presence or absence of the genes encoding GlgC and GlgB in the genomes of these species.

The TBLASTN results (Table 3) revealed that Pdp11, *S*. *putrefaciens* (taxid:24), and *S*. *algae* (taxid:38,313) possess conserved sequences with over 90% identity and complete coverage. In contrast, the genome of *S*. *hafniensis* (taxid: 365,590) did not contain any significant matches for *glgC and glgB*, suggesting the absence of these genes in the species.

**Table 3:** TBLASTN results showing the best matches in terms of alignment length, identity and coverage for the GlgC and GlgB proteins against the genome sequence of Pdp11, as well as all available strains of *S*. *putrefaciens*, *S*. *algae* and *S*. *hafniensis* in the NCBI database

Strains or NCBI genus *	Length		Identity (%)		Coverage (%)	
	*glgC*	*glgB*	*glgC*	*glgB*	*glgC*	*glgB*
***Shewanella* sp. Pdp11**	420	745	90.00	86.81	100	100
*Shewanella putrefaciens* (taxid:24) *	420	745	93.57	99.06	100	100
*Shewanella algae* (taxid:38,313) *	422	745	85.58	62.43	100	97
*Shewanella hafniensis* (taxid:365,590) *	No significant found					

### Effect of Exogenous Glycogen on Biofilm Formation in Shewanella Strains

To replicate the changes observed in biofilm formation upon exposure to Pdp11 FM2324 ECPs (Fig. 2) and to investigate whether glycogen is a metabolite involved in biofilm development, we conducted an assay using exogenous glycogen. In this study, the internal control of FM2324 (IC FM2324) mixed with TSBs was used as the base medium, to 2% glycogen (gly) and 1% raffinose (raf) were added, simulating the conditions of the probiotic FM2324 ECPs.

**Fig. 5:**
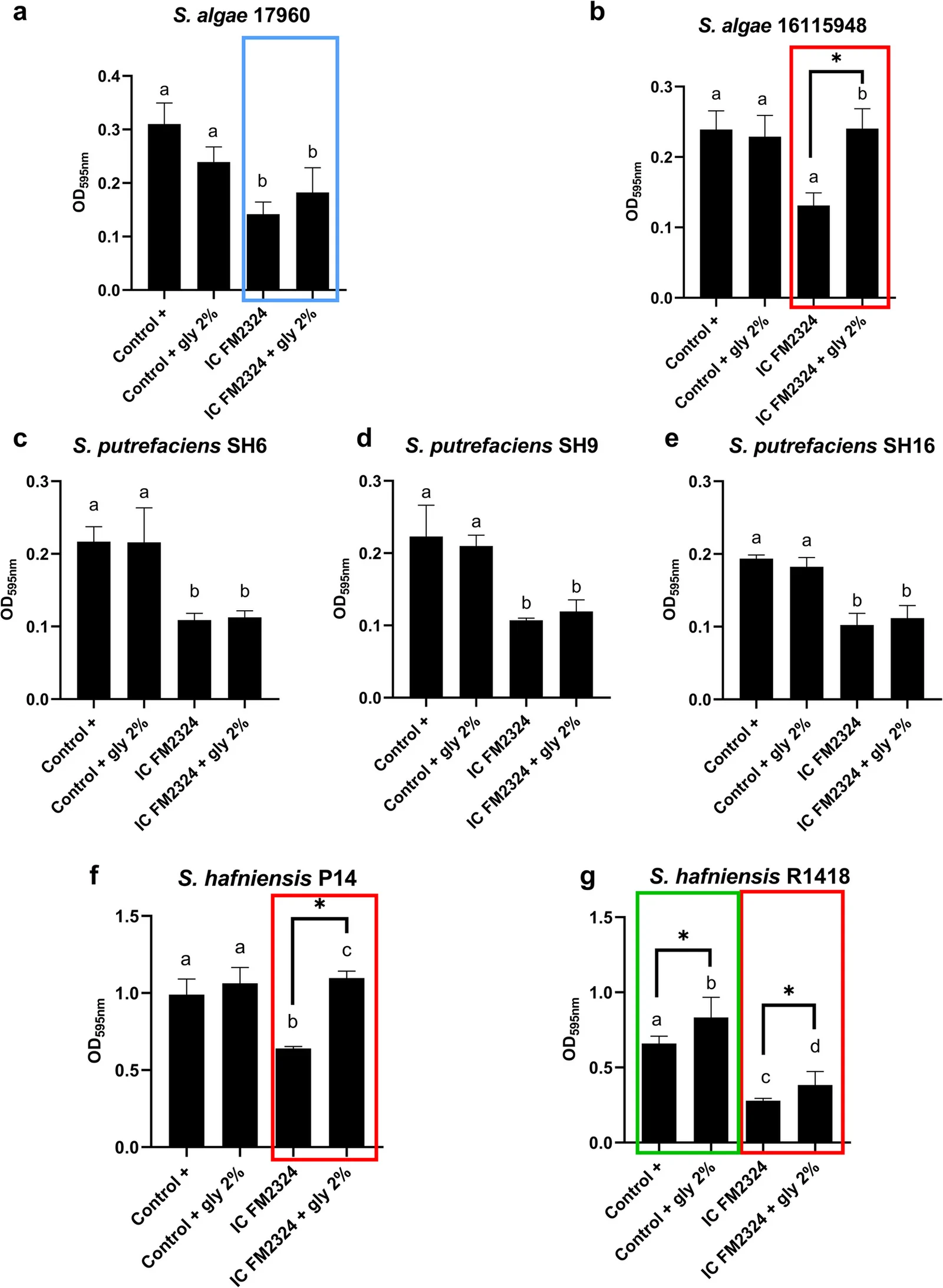
Biofilm formation of *S*. *hafniensis* P14 in contact with glycogen (2%). The Control + represents the bacterial biofilm formation in standard TSBs medium at 23 °C for 24 h without IC or glycogen. The results are expressed as mean ± SD (*n* = 5). Statistical analysis was carried out by one-way ANOVA. The letter (**a**, **b**, **c**, and **d**) indicates significant differences (*P* value < 0.05) between conditions analysed and the control of biofilm formation (Control +). A square with * indicates significant differences in biofilm formation by the absence or presence of exogen glycogen

The effect of exogenous glycogen on biofilm formation was then evaluated (Fig. 5) in the *Shewanella* strains using the CV method. Under normal bacterial culture conditions (TSBs, 23 ℃ and 24 h), serving as a positive control for biofilm formation (Control +), results indicated that no significant increase in *S*. *putrefaciens* biofilm formation was observed when exposed to exogenous glycogen. Similarly, *S*. *algae* 17,960 biofilm formation was not significantly influenced by glycogen, although a tendency to increase was observed (Fig. 5(a), blue box).

In contrast, *S*. *hafniensis* P14, *S*. *hafniensis* R1418 and *S*. *algae* 16,115,948 exhibited increased biofilm growth after supplementing the IC FM2324 with 2% glycogen, compared to IC FM2324 alone (Fig. 5(b, f, g), red box). The *S*. *hafniensis* P14 biofilm did not increase in contact with exogenous glycogen (Control + gly 2%). In contrast, biofilm was exposed to internal control (IC FM2324), which was reduced. Exogen glycogen (IC FM2324 + gly) countered this internal control effect. Additionally, in the case of *S*. *hafniensis* R1418, when the positive control was supplemented with exogenous glycogen, biofilm formation was induced, leading to a significant increase in biofilm values compared to its biofilm formation control (Fig. 5(g), green box).

The assays using the oligosaccharide raffinose 1% (Supplementary Fig. S3) did not reveal significant changes in biofilm formation. However, a slight attenuation of the inhibitory effect of IC FM2324 was observed in *S*. *putrefaciens*, *S*. *algae* 16,115,948 and *S*. *hafniensis* R1418, along with a reduction in biofilm formation in the control groups of all pathogenic *S*. *putrefaciens* strains. These changes, however, were not statistically significant.

## Discussion

In this study, we investigated the effects of extracellular products (ECPs) derived from Pdp11 on biofilm formation across multiple *Shewanella* strains, combining biofilm assays, metabolomic profiling, and genetic analyses to explore the underlying mechanisms. Our findings demonstrate a strain-dependent modulation of biofilm formation mediated by Pdp11 ECPs, highlighting glycogen as a key metabolite implicated in biofilm synthesis and structure.

Multiple factors can influence bacterial biofilm formation, including nutrient availability and ecological niche. Another important factor to consider when explaining differences in biofilm is strain’s host and origin of isolation [40]. The *S*. *putrefaciens* and *S*. *algae* were isolated from eel and human ulcers (open wounds), highlighting their roles as opportunistic pathogens in both fish and humans [8, 22]. In contrast, *S*. *hafniensis* strains were isolated from the healthy skin of cod and flounder [23], which suggests that their biofilm formation is associated with adhesion and survival strategies.

The results from CV staining assays revealed that IC derived from culture conditions influenced biofilm formation differently. These findings suggest that small-sized metabolites derived from the culture medium, capable of diffusing through cellophane, alter biofilm formation in all analyzed *Shewanella* strains, potentially related to supplementation with microalgae and cyanobacteria [21, 41]. This observation aligns with previous studies highlighting the antibacterial and antibiofilm properties of microalgal compounds in the aquaculture industry [42]. Interestingly, this effect was neither significantly enhanced nor diminished when using ECPs. Specifically, FM2324 significantly increased biofilm formation in *S*. *hafniensis* and *S*. *algae* strains. The strain-specific responses observed may be attributed to variations in the metabolic profiles of ECPs under different culture conditions, as demonstrated by metabolomic analysis. These findings are consistent with previous reports indicating that bacterial metabolites, including sugars and amino acids, can influence biofilm development by modulating extracellular polymeric substance production and structural organization [43, 44].

Microscopy analyses further confirmed that Pdp11 ECPs altered the structure and morphology of biofilms. *S*. *hafniensis* P14 biofilms exposed to FM2324, exhibited a denser and more cohesive extracellular matrix compared to the IC condition, as observed by confocal and SEM. These structural changes were accompanied by an increase in bacterial count and cell length, suggesting that Pdp11 ECPs not only influence biofilm quantity but also affect spatial organization and matrix density. Similar effects have been reported for other biofilm-promoting metabolites, including extracellular polysaccharides and proteins, which enhance bacterial adhesion and resilience [45, 46]. These results suggest that Pdp11 ECPs help preserve bacterial morphology, including length and count, essential for maintaining biofilm integrity. Thus, Pdp11-derived postbiotics reduce structural gaps and enhance matrix density, stabilizing biofilm architecture under specific conditions.

Metabolomic profiling provided insights into the composition of Pdp11 ECPs, identifying 23 metabolites exclusive to Pdp11 ECPs, including 14 enriched in FM2324, 2 in FM1548 and 7 common. Notably, glycogen emerged as a key metabolite with the most significant changes in abundance under FM2324 conditions. Glycogen has previously been implicated in biofilm formation, serving as an energy reserve and precursor for polysaccharide synthesis [47, 48]. The enrichment of glycogen in FM2324 ECPs correlated with increased biofilm formation, particularly in *S*. *hafniensis* strains, suggesting a metabolite-driven mechanism for biofilm modulation [49].

Genetic analysis further supports the role of glycogen in biofilm formation. TBLASTN searches identified conserved genes encoding glycogen biosynthesis enzymes [34, 50], such as *glgC* and *glgB*, in *S*. *putrefaciens* and *S*. *algae* strains. However, *S*. *hafniensis* genomes lacked homologs *glgC* and *glgB*, suggesting an inability to synthesize glycogen de novo. Although *glgC* and *glgA* knockouts have been reported to result in similar loss-of-function phenotypes [34], other studies indicate that *glgC* is more conserved and less variable across species. This gene encodes the first essential enzyme in the glycogen biosynthesis pathway [51, 52].

This genomic deficiency may explain the reliance of *S*. *hafniensis* on exogenous glycogen provided by Pdp11 ECPs to enhance biofilm development. Although *S*. *hafniensis* lacks the *glgC* and *glgB* genes, this does not entirely preclude the possibility that it may synthesize glycogen via alternative metabolic pathways, such as those involving trehalose metabolism [53, 54].

Exogenous supplementation with raffinose, a metabolite know to modulate biofilm formation in other bacterial genera [38, 39]. In contrast, the addition of glycogen to the culture medium reinforced this observation by promoting biofilm development in *S*. *hafniensis* P14 and R1418, as well as in *S*. *algae* 16,115,948—mirroring the effects observed with ECPs from Pdp11. These effects mirrored those observed with Pdp11 ECPs, reinforcing the role of glycogen as a biofilm-promoting factor. This aligns with previous studies demonstrating that glycogen facilitates biofilm maturation by supporting the synthesis of structural matrix components and enhancing bacterial adhesion [55].

However, the functional consequences of this genetic deficiency have not been experimentally validated. Further studies incorporating functional approaches, such as knockout mutants or complementation assays, are necessary to confirm these genetic inferences and elucidate their role in biofilm formation. Previous studies have shown that glycogen serves as an energy reserve for bacteria, facilitating survival during nutrient deprivation and enhancing biofilm stability [56–58]. The accumulation of glycogen in Pdp11 ECPs may contribute to biofilm resilience in *Shewanella* species, particularly those lacking endogenous glycogen biosynthesis pathways.

Although *S*. *hafniensis* P14 and R1418 exhibit similar phenotypic and biochemical characteristics [23], and respond similarly to exogenous glycogen, P14 consistently forms denser biofilms (OD595 ~ 1), approximately double that of R1418. This difference represents a key phenotypic distinction between the two strains.

Interestingly, glycogen did not further increase biofilm biomass in *S*. *hafniensis* P14 under standard (Control +) conditions, likely due to a saturation effect, as supported by microscopy (Fig. 3a, Supplementary Fig. S2). However, in the presence of IC—which introduces inhibitory metabolites—glycogen supplementation partially restored biofilm structure and density. This suggests that glycogen may act less as a universal biofilm promoter and more as a compensatory factor, mitigating the impact of inhibitory compounds in the environment. These strain- and context-specific effects underscore the complexity of biofilm regulation and suggest that the activity of glycogen is dependent on both the inherent biofilm-forming capacity of the strain and external environmental pressures.

The behavior of *S*. *hafniensis* P14, in particular, raises questions about its ecological adaptation and metabolic flexibility. Its inability to synthesize glycogen may represent a metabolic trade-off, relying instead on environmental sources to support biofilm development [59]. This dependency could provide competitive advantages in nutrient-rich environments, where extracellular glycogen becomes accessible through interactions with other microbial communities, such as Pdp11. Alternatively, this trait might enhance adaptability to fluctuating nutrient conditions by minimizing the metabolic cost associated with glycogen biosynthesis [60]. Future studies could explore whether this dependency influences biofilm stability, resilience, or dispersal, particularly in multispecies biofilm systems, offering insights into microbial interactions and niche specialization.

Despite these promising results, several limitations must be acknowledged. First, this study focused primarily on soluble metabolites, leaving non-soluble and intracellular metabolites unexplored. This presents a partial view of the bioactive components involved in biofilm modulation. Additionally, our results underscore the biological and applied relevance of temperature-dependent variation in ECP composition, particularly in the context of optimizing postbiotic production for aquaculture applications. Future research should incorporate targeted metabolomics to capture a more comprehensive profile of biofilm-modulating compounds. Second, while glycogen emerged as a central metabolite, the contributions of other identified compounds, such as taurine and d-erythrose, remain unclear and warrant further investigation. Taurine, for instance, has been associated with antioxidant and anti-biofilm activities [61], raising the possibility of synergistic interactions between metabolites in shaping biofilm dynamics.

Our findings have broader implications for the development of metabolite-based strategies to control biofilms in aquaculture and industrial applications. The differential response of *Shewanella* strains to Pdp11 ECPs suggests that metabolite-mediated biofilm modulation plays a role in microbial succession and niche adaptation in aquatic environments. This mechanism may influence community composition, particularly in biofilm-dominated ecosystems such as aquaculture systems, marine sediments, and other submerged surfaces.

Notably, Pdp11 ECPs, enriched in biofilm-promoting metabolites, could be leveraged to enhance beneficial biofilms that outcompete pathogenic species or to stabilize biofilms in biofilter systems. Conversely, elucidating the mechanisms through which ECPs drive biofilm formation may inform the design of anti-biofilm interventions targeting specific metabolite pathways. For instance, disrupting glycogen metabolism could provide novel biofilm control strategies without relying on antibiotics, thus mitigating the risk of antimicrobial resistance [62].

In conclusion, this study highlights the dual role of Pdp11 ECPs as biofilm modulators, with strain-specific responses driven by metabolite composition. Glycogen emerged as a key factor influencing biofilm formation, particularly in strains lacking the genetic machinery for glycogen biosynthesis. These insights contribute to our understanding of metabolite-mediated biofilm regulation and underscore the potential of postbiotics as tools for biofilm control in aquaculture and industrial settings. Further research is needed to elucidate the molecular mechanisms underlying metabolite-biofilm interactions and to evaluate the scalability of these findings for practical applications.

## Supplementary Information

Below is the link to the electronic supplementary material.

ESM 1 (DOCX 878 KB)

ESM 2 (DOCX 2.93 MB)

ESM 3 (DOCX 223 KB)

ESM 4 (DOCX 16.5 KB)

ESM 5 (FASTA 8.72 KB)

ESM 6 (FASTA 6.54 KB)

## Data Availability

Untargeted metabolomics raw data files and metadata are available in the public Mass Spectrometry Interactive Virtual Environment (MASSIVE) (https://massive.ucsd.edu/ProteoSAFe/static/massive.jsp), Dataset Identifiers: MSV000095402.
