# Supplementary material for: Metabolite-Driven Modulation of Biofilm Formation in Shewanella: Insights from Shewanella sp. Pdp11 Extracellular Products
Source: Microb Ecol. 2025 May 27;88:55. doi: 10.1007/s00248-025-02552-x (PMC12116997; doi:10.1007/s00248-025-02552-x)
Supplement: Supplementary file 1 [file 248_2025_2552_MOESM1_ESM.docx]

**Supplementary material**

**Journal name: Microbial Ecology**

**Research article:** Metabolite-driven modulation of biofilm formation in *Shewanella*: insights from *Shewanella* sp. Pdp11 extracellular products

Olivia, Pérez-Gomez^1^, Marta Domínguez-Maqueda^1^, Jorge García-Márquez^1^, Miguel Ángel Moriñigo^1^, Silvana T. Tapia-Paniagua^1^

^1^Department of Microbiology, Faculty of Sciences, University of Malaga, Málaga, Spain

**Corresponding authors: Silvana T. Tapia-Paniagua (stapia@uma.es)**

**Fig. S1** Bacterial growth curves for each strain (a–g) after 24 hours of incubation with extracellular products (ECPs) from *Shewanella* sp. Pdp11 under different conditions. The y-axis represents optical density (OD), while the x-axis shows the concentration of ECPs (F2324, M2324, FM2324, FM1548) along with their respective internal controls. Growth differences between the positive control, internal control (IC), and Pdp11 ECPs were assessed using one-way ANOVA followed by Dunnett’s multiple comparisons test in GraphPad Prism 8.0.1 (P < 0.05). The minimum inhibitory concentration (MIC) is indicated by a red arrow, while the lower concentrations used in biofilm formation assays are marked with a blue arrow—at these concentrations, no significant differences were observed between the control, IC, and ECPs.
