# Supplementary material for: Metabolite-Driven Modulation of Biofilm Formation in Shewanella: Insights from Shewanella sp. Pdp11 Extracellular Products
Source: Microb Ecol. 2025 May 27;88:55. doi: 10.1007/s00248-025-02552-x (PMC12116997; doi:10.1007/s00248-025-02552-x)
Supplement: Supplementary file 3 [file 248_2025_2552_MOESM3_ESM.docx]

**Supplementary material**

**Journal name: Microbial Ecology**

**Research article:** Metabolite-driven modulation of biofilm formation in *Shewanella*: insights from *Shewanella* sp. Pdp11 extracellular products

Olivia, Pérez-Gomez^1^, Marta Domínguez-Maqueda^1^, Jorge García-Márquez^1^, Miguel Ángel Moriñigo^1^, Silvana T. Tapia-Paniagua^1^

^1^Department of Microbiology, Faculty of Sciences, University of Malaga, Málaga, Spain

**Corresponding authors: Silvana T. Tapia-Paniagua (stapia@uma.es)**

**Fig. S3** Biofilm formation of *S. hafniensis* P14 in contact with raffinose (1%). The positive control represents the standard bacterial biofilm formation in TSBs at 23 °C for 24 hours, while IC FM2324 serves as the internal control for each ECP. The results are expressed as mean ± SD (n = 5). Statistical analysis was carried out by one-way ANOVA. The letter (a, b, c, and d) indicates significant differences (P-value < 0.05) between conditions analysed and the control of biofilm formation (Control +). A square with * indicates significant differences in biofilm formation by the absence or presence of exogen raffinose.
