# Supplementary material for: Metabolite-Driven Modulation of Biofilm Formation in Shewanella: Insights from Shewanella sp. Pdp11 Extracellular Products
Source: Microb Ecol. 2025 May 27;88(1):55. doi: 10.1007/s00248-025-02552-x (PMC12116997; doi:10.1007/s00248-025-02552-x)
Supplement: Supplementary file 2 — (DOCX 2.93 MB) [file 248_2025_2552_MOESM2_ESM.docx]

**Supplementary material**

**Journal name: Microbial Ecology**

**Research article:** Metabolite-driven modulation of biofilm formation in *Shewanella*: insights from *Shewanella* sp. Pdp11 extracellular products

Olivia, Pérez-Gomez^1^, Marta Domínguez-Maqueda^1^, Jorge García-Márquez^1^, Miguel Ángel Moriñigo^1^, Silvana T. Tapia-Paniagua^1^

^1^Department of Microbiology, Faculty of Sciences, University of Malaga, Málaga, Spain

**Corresponding authors: Silvana T. Tapia-Paniagua (stapia@uma.es)**


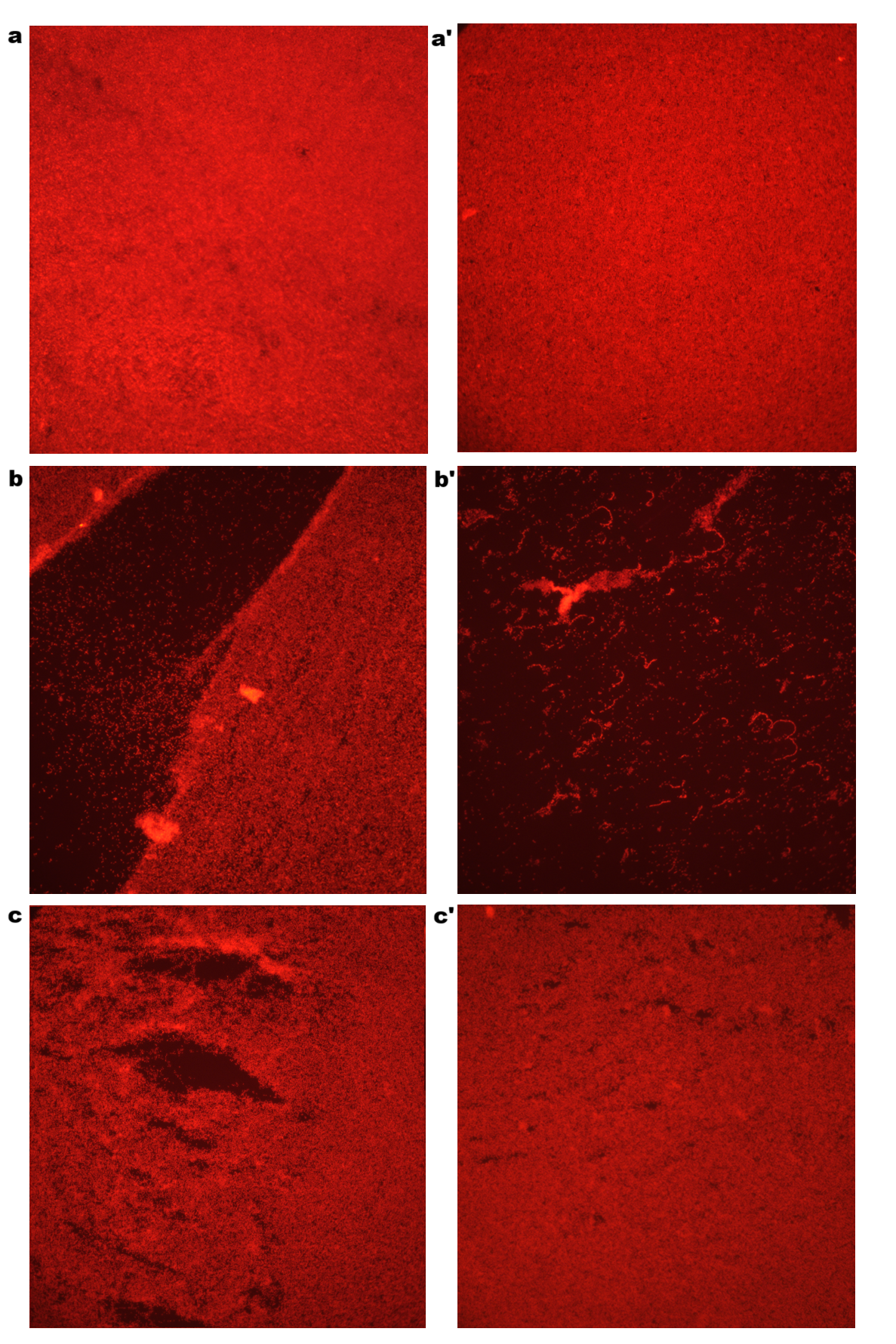


**Fig. S2** Biofilm architecture of *S. hafniensis* P14 observed via fluorescence microscopy under different conditions: untreated control (a, a'), exposure to the internal control (IC) derived from FM2324 (b, b'), and treatment with extracellular products (ECPs) from *Shewanella* sp. Pdp11 (FM2324) (c, c'). All samples were incubated at 23 °C for 24 hours.
