# Supplementary material for: Metabolite-Driven Modulation of Biofilm Formation in Shewanella: Insights from Shewanella sp. Pdp11 Extracellular Products
Source: Microb Ecol. 2025 May 27;88(1):55. doi: 10.1007/s00248-025-02552-x (PMC12116997; doi:10.1007/s00248-025-02552-x)
Supplement: Supplementary file 4 — (DOCX 16.5 KB) [file 248_2025_2552_MOESM4_ESM.docx]

**Supplementary material**

**Journal name: Microbial Ecology**

**Research article:** Metabolite-driven modulation of biofilm formation in *Shewanella*: insights from *Shewanella* sp. Pdp11 extracellular products

Olivia, Pérez-Gomez^1^, Marta Domínguez-Maqueda^1^, Jorge García-Márquez^1^, Miguel Ángel Moriñigo^1^, Silvana T. Tapia-Paniagua^1^

^1^Department of Microbiology, Faculty of Sciences, University of Malaga, Málaga, Spain

**Corresponding authors: Silvana T. Tapia-Paniagua (stapia@uma.es)**

**Table S1.** Minimum inhibitory concentration (MIC) is established for each strain in contact to ECP of *Shewanella*. sp Pdp11 after 24h of incubation with different ECP conditions (µg protein/µl).

| Strains | Pdp11 ECPs protein concentrations (µg/µl) | | | |
| --- | --- | --- | --- | --- |
|  | F2324 | M2324 | FM2324 | FM1548 |
| *S. putrefaciens* SH6 | 0.003 | 0.025 | 0.100 | 0.013 |
| *S. putrefaciens* SH9 | 0.013 | 0.025 | 0.100 | 0.025 |
| *S. putrefaciens* SH16 | 0.013 | 0.100 | 0.100 | 0.050 |
| *S. algae* 17960 | 0.013 | 0.013 | 0.025 | 0.025 |
| *S. algae* 16115948 | 0.003 | 0.013 | 0.100 | 0.006 |
| *S. hafniensis* P14 | 0.025 | 0.050 | 0.100 | 0.050 |
| *S. hafniensis* R1418 | 0.025 | 0.050 | 0.050 | 0.050 |
